# Targeting Interleukin-4 Receptor Alpha by Hybrid Peptide for Novel Biliary Tract Cancer Therapy

**DOI:** 10.1155/2014/584650

**Published:** 2014-04-27

**Authors:** Kahori Seto, Junichi Shoda, Tomohisa Horibe, Eiji Warabi, Masayuki Kohno, Toru Yanagawa, Hiroki Bukawa, Yasuni Nakanuma, Koji Kawakami

**Affiliations:** ^1^Department of Oral and Maxillofacial Surgery, Clinical Sciences, University of Tsukuba, Ibaraki 305-8575, Japan; ^2^Department of Pharmacoepidemiology, Graduate School of Medicine and Public Health, Kyoto University, Yoshida Konoe-cho, Sakyo-ku, Kyoto 606-8501, Japan; ^3^Medical Science, Faculty of Medicine, University of Tsukuba, Ibaraki 305-8575, Japan; ^4^Division of Biomedical Science, Faculty of Medicine, University of Tsukuba, Ibaraki 305-8575, Japan; ^5^Department of Human Pathology, Graduate School of Medical Science, Kanazawa University, Kanazawa 920-1192, Japan

## Abstract

It is known that the interleukin-4 receptor **α** (IL-4R**α**) is highly expressed on the surface of various human solid tumors. We previously designed novel IL-4R**α**-lytic hybrid peptide composed of binding peptide to IL-4R**α** and cell-lytic peptide and reported that the designed IL-4R**α**-lytic hybrid peptide exhibited cytotoxic and antitumor activity both *in vitro* and *in vivo* against the human pancreatic cancer cells expressing IL-4R**α**. Here, we evaluated the antitumor activity of the IL-4R**α**-lytic hybrid peptide as a novel molecular targeted therapy for human biliary tract cancer (BTC). The IL-4R**α**-lytic hybrid peptide showed cytotoxic activity in six BTC cell lines with a concentration that killed 50% of all cells (IC_50_) as low as 5 **μ**M. We also showed that IL-4R**α**-lytic hybrid peptide in combination with gemcitabine exhibited synergistic cytotoxic activity *in vitro*. In addition, intravenous administration of IL-4R**α**-lytic hybrid peptide significantly inhibited tumor growth in a xenograft model of human BTC *in vivo*. Taken together, these results indicated that the IL-4R**α**-lytic hybrid peptide is a potent agent that might provide a novel therapy for patients with BTC.

## 1. Introduction

Biliary tract cancer such as gallbladder cancer and extrahepatic bile duct cancer as well as intrahepatic bile duct cancer (one of the primary liver cancers) is likely to undergo metastasis to the peritoneum (peritoneal dissemination) or the liver at early stages and is often resistant to conventional chemotherapy and radiotherapy. These cancers have been thus viewed as intractable cancers unlikely to be cured completely. In Japan, the incidence of biliary tract cancer and intrahepatic bile duct cancer is about 10 out of every 100,000 people [1]. As for intrahepatic bile duct cancer, both the incidence and death rate have been rising in Japan in recent years, resembling the trend observed in western countries [2, 3]. In Japan, gemcitabine and S-1 have recently begun to be used for anticancer chemotherapy, and these drugs are expected to prolong the survival period of patients as compared to existing anticancer drugs [4]. However, because of frequent adverse events of the hematological system arising from these drugs and because of compromised hepatic function often noted in patients with intrahepatic bile duct cancer due to accompanying liver cirrhosis and in those with extrahepatic bile duct cancer or gallbladder cancer due to accompanying obstructive cholestasis, treatment with these drugs has to be discontinued or stopped. To improve the outcome of treatment of these cancers, it is very important to identify the tumor biological factors involved in the progression (e.g., infiltration and metastasis) of the cancers and to develop new valid means of auxiliary treatment targeted at these factors.

Based on the immunohistochemical analysis showing that cultured BTC cell lines and cancerous epithelia in BTC tissue expressed receptors for interleukin-4 (IL-4)* in situ* at high densities, we previously tested the antitumor effects of IL-4-PE, a cytotoxin composed of an interleukin-4 (IL-4) targeting moiety and a truncated form of* Pseudomonas* exotoxin, on human BTC cells [5]. The results showed a significant regression of established BTC tumors and a significant prolongation of the survival of animals with the disseminated tumors [5, 6]. IL-4-PE was previously tested in the clinic for the treatment of human solid tumors [7–10]. However, against expectation of these promising results, the clinical application of IL-4-PE faced many challenges, including nonspecific toxicities and immunogenicity [11].

To overcome these issues, we have recently developed a “hybrid peptide,” composed of target-binding and cytotoxic sequences containing cationic-rich D- and L-amino acids to form amphipathic partial *α*-helices that disrupt the cancer cell membrane selectively and are stable when combined with a cancer-targeting moiety [12]. Peptide drugs are relatively easily synthesized using either recombinant or solid-phase chemical synthesis techniques and the production costs are generally affordable when compared to antibody-based therapeutics [12]. The hybrid peptides targeting EGFR, transferrin receptor, Hsp90, neuropilin-1, and interleukin-4 receptor *α* (IL-4R*α*) have shown cytotoxic activity* in vitro* and antitumor activity* in vivo* against human solid tumors [12–17]. The IL-4R*α*-lytic hybrid peptide is composed of an IL-4R*α*-binding moiety and a cellular-membrane-lytic moiety [17]. Although the biological significance of IL-4R*α* expression of cancer cells remains unclear, this receptor is a candidate applicable for molecular targeted cancer therapy.

Here, we describe the cytotoxic activity by IL-4R*α*-lytic hybrid peptide toward BTC cell lines which overexpress IL-4R*α in vitro*. We also show the synergetic effect of IL-4R*α*-lytic hybrid peptide with gemcitabine against BTC cells i*n vitro* and that this hybrid peptide induces the significant regression of established human xenograft BTC tumor model in mouse* in vivo*.

## 2. Materials and Methods

### 2.1. Cell Lines and Culture Conditions

The experiments were performed on 4 human cholangiocarcinoma cell lines, KMBC [18] from Dr. M. Kojiro (Kurume University School of Medicine, Kurume, Japan), Sk-ChA-1 [19] from Dr. A. Knuth (Johannes Gutenberg University, Mainz, Germany), CCKS-1 [20] from Dr. Y. Nakanuma (Kanazawa University Graduate School, Kanazawa, Japan), and KKU-100 [21, 22] from Dr. B. Sripa (Khon Kaen University, Thailand), and on 2 human gallbladder carcinoma cell lines: TGBC-1-TKB and TGBC-44-TKB [23] from Dr. T. Todoroki (University of Tsukuba, Ibaraki, Japan). All BTC cells were cultured in RPMI 1640 medium containing 10% heat-inactivated fetal calf serum (Invitrogen, Carlsbad, CA, USA) in a humidified atmosphere with 5% CO_2_ at 37°C.

### 2.2. Peptides

IL-4R*α*-lytic hybrid peptide, KQLIRFLKRLDRNGGGKL**L**LK**L**L**KK**LLK**L**LKKK (bold and underlined letters correspond to D-amino acids), and lytic peptide, KL**L**LK**L**L**KK**LLK**L**LKKK, were synthesized by Invitrogen (Carlsbad, CA, USA). Peptides were dissolved in water and buffered to pH 7.4 as described previously [17].

### 2.3. Immunoblot Analysis

Immunoblot analysis was carried out as described previously [5]. Briefly, whole-cell extracts were obtained using buffer containing 1% (v/v) Triton X-100, 0.1% (w/v) SDS, and 0.5% (w/v) sodium deoxycholate, separated by SDS/PAGE, and transferred onto a PVDF membrane. IL-4R*α* antibody was used at dilution 1 : 100 (Santa Cruz Biotechnology, CA, USA) and *β*-actin was used as the internal control (R&D Systems, Minneapolis, MN, USA). After treatment with horseradish peroxidase-conjugated anti-mouse IgG antibody, proteins were visualized on Hyperfirm using an enhanced chemiluminescence/western blotting system (GE Healthcare, Piscataway, NJ, USA).

### 2.4. Cell Viability Assay

Cell viability assay was performed as described previously [12]. Briefly, cells were seeded into 96-well plates at 3 × 10^3^ cells per well in 90 *μ*L medium and incubated at 37°C for 24 hours. Each peptide (IL-4R*α*-lytic hybrid peptide and lytic peptide) was diluted in 10 *μ*L culture medium and added to the cells. The concentration range of peptide we used was from 0 to 20 *μ*M. After 72 hours of incubation, the cell viability assay using WST-8 solution (Nacalai Tesque, Kyoto, Japan) was performed.

### 2.5. Combination Assay of Peptide and Gemcitabine

The* in vitro* cytotoxic activity of IL-4R*α*-lytic hybrid peptide and gemcitabine was measured as cell viability assay. The synergistic effect of IL-4R*α*-lytic hybrid peptide and gemcitabine was assessed at a concentration ratio of 1 : 1, using the combination index (CI), where CI < 1, CI = 1, and CI > 1 indicate synergistic, addictive, and antagonistic effects, respectively. The CI value was calculated as described previously [24].

### 2.6. Antitumor Activity of IL-4R*α*-Lytic Hybrid Peptide in Xenografted Tumor Model of Human BTC 

Animal experiments were carried out in accordance with the guidelines of Tsukuba University. 1 × 10^6^ TGBC-44-TKB cells resuspended in 150 *μ*L phosphate-buffered saline were inoculated subcutaneously into the flank region of 4- to 6-week-old athymic female nude mice weighing 17–20 g. When the tumor reached 20–60 mm^3^ in volume, the animals were assigned randomly to 3 groups: saline (control) and IL-4R*α*-lytic hybrid peptide (2 or 5 mg/kg) injected intravenously (50 *μ*L/injection) three times a week for a total of nine times. Each group had 6 animals (*n* = 6). Tumors were measured with a caliper and the tumor volume (in mm^3^) was calculated using the formula length × width^2^ × 0.5.

### 2.7. Statistics

Values were given as mean ± SD (standard deviation), and the differences were analyzed using a one-way ANOVA with Dunnett's test. Differences were considered to be statistically significant at *P* < 0.05.

## 3. Results

### 3.1. Expression of IL-4R*α* in BTC Cell Lines

**Figure 1:**
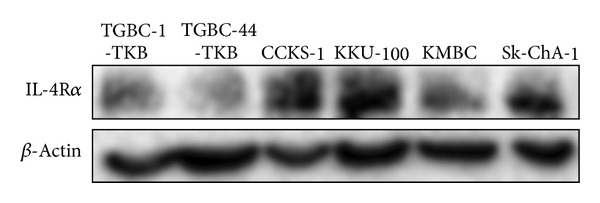
Expression levels of IL-4R*α* in BTC cells. Cell lysates were prepared from six BTC cell lines. *β*-Actin was used as an internal control.

Immunoblot analysis demonstrated that IL-4R*α* protein is expressed in all cultured BTC cell lines used in this study. As shown in Figure 1, the expression levels of IL-4R*α* protein were the highest in intrahepatic cholangiocarcinoma cell lines (CCKS-1 and KKU-100) among tested cell lines. On the other hand, it was also confirmed that gallbladder carcinoma cell lines (TGBC-1-TKB and TGBC-44-TKB) and extrahepatic cholangiocarcinoma cell lines (KMBC and Sk-ChA-1) expressed IL-4R*α*, although the expression levels were lower than those of CCKS-1 and KKU-100 cells.

### 3.2. Cytotoxic Activity of IL-4R*α*-Lytic Hybrid Peptide in BTC Cell Lines

**Figure 2:**
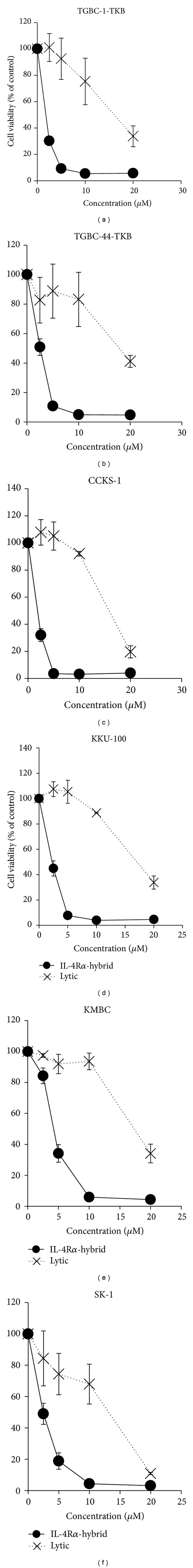
Cytotoxic activity of IL-4R*α*-lytic hybrid peptide. Six BTC cell lines were cultured with various concentrations of IL-4R*α*-lytic hybrid peptide or lytic peptide (0–20 *μ*M) for 72 h, and cytotoxic activity was assessed using WST-8 reagent. The results are represented as mean ± SD (bars) of triplicate determinations, and the assay was repeated three times.

To assess the* in vitro* cytotoxic activity of IL-4R*α*-lytic hybrid peptide to BTC cell lines, the WST assay was performed using BTC cell lines treated with IL-4R*α*-lytic hybrid peptide and lytic peptide (Figure 2). TGBC-1-TKB, TGBC-44-TKB, CCKS-1, KKU-100, and KMBC were sensitive to IL-4R*α*-lytic hybrid peptide; the concentration that killed 50% of all cells (IC_50_) was less than 3.5 *μ*M. Sk-ChA-1 was also sensitive to IL-4R*α*-lytic hybrid peptide with an IC_50_ of less than 4.5 *μ*M. In contrast, optimal cell killing was not induced in these cells by lytic peptide. The cytotoxic activity of the hybrid peptide was strongly enhanced when compared with that of the lytic peptide. The cytotoxic activity of IL-4R*α*-lytic hybrid peptide increased 3.0–7.8-fold stronger than that of lytic peptide in BTC cell lines (Table 1).

### 3.3. Synergistic Cytotoxicity of IL-4R*α*-Lytic Hybrid Peptide and Gemcitabine in BTC Cells

**Figure 3:**
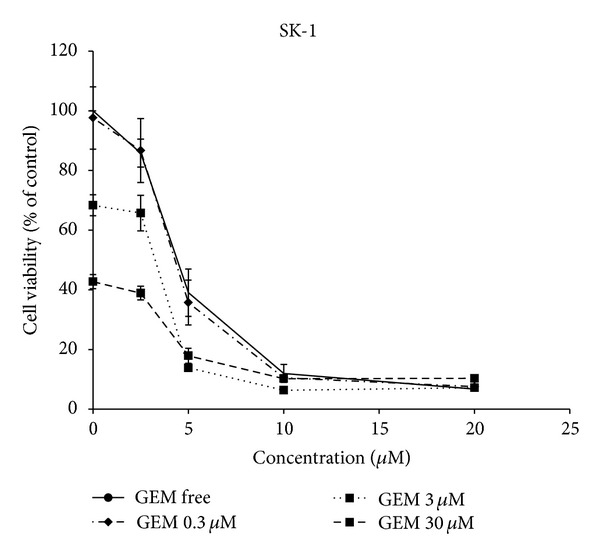
Cytotoxic activity by combination therapy of IL-4R*α*-lytic hybrid peptide with gemcitabine. SK-1 was incubated with various concentrations of IL-4R*α*-lytic hybrid peptide (0–20 *μ*M) and gemcitabine (0–30 nM). The results are represented as mean ± SD (bars) of triplicate determinations, and the assay was repeated three times.

Gemcitabine alone mediated a dose-dependent cytotoxicity with IC_50_ of 12 nM in Sk-ChA-1. When it combined with IL-4R*α*-lytic hybrid peptide, cytotoxicity in Sk-ChA-1 was greatly enhanced (Figure 3). IC_50_ of IL-4R*α*-lytic hybrid peptide was 4.29 and 3.25 *μ*M by adding 0.3 and 3 nM of gemcitabine, respectively (Table 2). The combination index was <1 at all concentrations of gemcitabine. The same phenomenon was also observed in TGBC-44-TKB (data not shown).

### 3.4. *In Vivo* Antitumor Activity by IL-4R*α*-Lytic Hybrid Peptide in a Human BTC Xenograft Model

**Figure 4:**
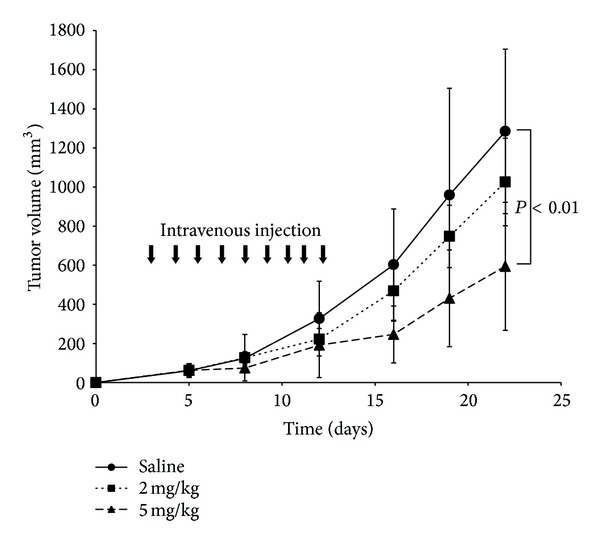
Antitumor activity of IL-4R*α*-lytic hybrid peptide in tumor xenograft model* in vivo*. TGBC-44-TKB were implanted subcutaneously into athymic nude mice. Intravenous injection of saline and IL-4R*α*-lytic hybrid peptide (2 mg/kg or 5 mg/kg) is indicated by the arrows. Data are expressed as mean ± SD (*n* = 6 animals in each group).

Following the observation that IL-4R*α*-lytic hybrid peptide was remarkably cytotoxic to BTC cell lines* in vitro* (Figure 2), the antitumor activity of the hybrid peptide was assessed in a xenograft model of human BTC. TGBC-44-TKB cells were inoculated subcutaneously into athymic nude mice and then the animals were treated with IL-4R*α*-lytic hybrid peptide by intravenous injection. As shown in Figure 4, tumors grew aggressively in control mice injected with saline alone, reaching a volume of 1285 mm^3^ by day 22. In contrast, mice treated with IL-4R*α*-lytic hybrid peptide showed significant tumor regression at both dosages: mean tumor volumes were 1025 mm^3^ (2 mg/kg) and 594 mm^3^ (5 mg/kg) on day 22. The tumor volume was inhibited significantly at a dose of 5 mg/kg (*P* < 0.05). No other abnormalities, such as loss of appetite and body weight, were observed in mice injected with IL-4R*α*-lytic hybrid peptide (data not shown). The number of leukocytes (the mean (×10^3^) and SD values) after the experiments is as follows: control group: 5.1 ± 2.1, 2 mg/kg group: 5.6 ± 4.0, and 5 mg/kg group: 6.6 ± 4.7, and there are no significant differences in each group. Histological analysis also showed no side effects in tissue from the major organs, including liver and kidney, which were obtained from mice treated with intravenous administration of IL-4R*α*-lytic hybrid peptide (data not shown). These results demonstrated that IL-4R*α*-lytic hybrid peptide induced an effective antitumor activity in a mouse xenograft model of BTC.

## 4. Discussion

Therapeutic options for BTC are unsatisfactory, and the survival outcome is therefore poor. Effective therapeutic approaches against this aggressive disease are urgently needed. We previously demonstrated that human BTC tissue specimens expressed high levels of IL-4 receptor compared to normal gallbladders [5]. These observations were also confirmed in cultured human BTC cell lines that expressed IL-4R by immunoblot analysis. Immunohistochemical analysis showed overexpression of IL-4R in a large proportion (50–63%) of human BTC tissue specimens. Following the results of preclinical study for treatment of BTC with IL-4-PE [5], the efficacy of IL-4R*α*-lytic hybrid peptide against human BTC was evaluated in preclinical models in this study.

Using the approach of selective receptor targeting, we have tested the efficacy of IL-4R*α*-lytic hybrid peptide on BTC cells. The overexpression of IL-4R*α* in BTC cell lines sensitized them to the cytotoxic effect of this peptide, which correlated with the level of IL-4R*α* expression. The* in vitro* cytotoxicity of this peptide was assessed for 6 cultured BTC cell lines (Figure 1). KMBC and CCKS-1 cell lines showing higher levels of IL-4R*α* expression showed higher sensitivity (IC_50_ of IL-4R*α*-lytic hybrid peptide toward these cancer cells was approximately 2 *μ*M, shown in Table 1). However the degree of the effect of IL-4R*α*-lytic hybrid peptide against each BTC cell line is hardly different. These observations indicate that abundant IL-4R*α* expression in BTC tumors will facilitate efficient targeting by this hybrid peptide. We previously reported that IL-4R*α*-lytic had effective cytotoxic activity toward cancer cells expressing IL-4R*α* and that enhancement of the IL-4R*α*-lytic peptide-induced cytotoxicity correlates well with the expression levels of IL-4R*α* [17]. We also previously showed the specificity of this hybrid peptide between normal and cancer cells by binding assay [25]. Moreover, it was demonstrated that intravenous administration of this hybrid peptide at 5 mg/kg dramatically inhibited the growth of TGBC44-TKB tumors* in vivo *(Figure 4). Histologic analysis also showed no abnormal changes in the tissue of major organs obtained from mice treated with the hybrid peptide (data not shown).

In this study, IL-4R*α*-lytic hybrid peptide in combination with gemcitabine exhibited synergistic cytotoxic activity* in vitro* (Figure 3). Gemcitabine (Gemzar) is a widely accepted first-line therapy for advanced BTC, although the median survival time is not favorable [26]. Most studies using a single agent showed a low response rate and little effect on patient survival in advanced adenocarcinoma. It has recently been reported that cisplatin plus gemcitabine is associated with a significant survival advantage as compared with gemcitabine alone [4]. Moreover, several clinical trials using a combined approach of molecular target therapy with gemcitabine and other drugs have been initiated [27, 28]. Previously, IL-4-PE was shown to exert a synergistic effect with gemcitabine against the* in vitro* and* in vivo* pancreatic cancer models [28]. The results of this study support our observations of gemcitabine synergizing with IL-4R*α*-lytic hybrid peptide on BTC cells, although the precise mechanism of the synergistic effect of gemcitabine with this hybrid peptide still remains obscure. It is known that gemcitabine inhibits DNA synthesis through inhibition of ribonucleotide reductase and depletion of deoxynucleotide pools [29]. It is suggested that the inhibition effect by gemcitabine in cancer cells might be exerted synergetically with the disintegration activity toward cancer cell membrane by IL-4R*α*-lytic hybrid peptide [12, 17]. Thus, it is possible that gemcitabine enhances cell death induced by IL-4R*α*-lytic hybrid peptide. Considering cancer cell death, the combination therapy of these drugs, which act through different mechanisms, may be a beneficial treatment option for patients with BTC.

## 5. Conclusions

Our current data describe that IL-4R*α* is overexpressed in BTC cell lines* in vitro*, and BTC cells which overexpress the receptor can be successfully targeted with IL-4R*α*-lytic hybrid peptide both* in vitro* and* in vivo*. IL-4R*α*-lytic hybrid peptide induces regression of the established mouse model of BTC tumor. Thus, the findings of this study will assist and are available for elucidation of novel and effective BTC therapy targeting IL-4R*α* by IL-4R*α*-lytic hybrid peptide.

## Figures and Tables

**Table 1:** Cytotoxic activity of IL-4R*α*-lytic hybrid peptide against BTC.

Cell line	IC_50_ (*μ*M)		IC_50_ ratio
	Lytic peptide alone	IL-4R*α*-lytic peptide	(Lytic/IL-4R*α*-lytic )
TGBC-1-TKB	16.99 ± 1.28	2.17 ± 0.54	7.83
TGBC-44-TKB	21.37 ± 4.91	3.27 ± 0.51	6.53
CCKS-1	15.83 ± 0.02	2.73 ± 1.26	5.80
KKU-100	18.93 ± 3.85	2.99 ± 1.01	6.34
KMBC	18.50 ± 1.65	3.25 ± 1.36	5.69
SK-1	13.44 ± 0.42	4.47 ± 0.40	3.00

IC_50_ values (peptide concentration inducing 50% inhibition of control cell growth) are represented as mean ± SD from triplicate determinations.

**Table 2:** Combination therapy of hybrid peptide and GEM.

	IC_50_	CI
IL-4R hybrid peptide (*μ*M)	4.47	
Gemcitabine (nM)	12.2	
IL-4R hybrid peptide (*μ*M) + gemcitabine 0.3 nM	4.29	0.95
IL-4R hybrid peptide (*μ*M) + gemcitabine 3 nM	3.25	0.75

CI (combination index) < 1, CI = 1, and CI > 1 indicate synergistic, addictive, and antagonistic effect, respectively.
